# Classical-Quantum Transition as a Disorder-Order Process

**DOI:** 10.3390/e24010087

**Published:** 2022-01-05

**Authors:** Andres M. Kowalski, Angelo Plastino

**Affiliations:** 1 Departamento de Física, Universidad Nacional de La Plata, La Plata 1900, Argentina; kowalski@fisica.unlp.edu.ar; 2 Comisión de Investigaciones Científicas (CICPBA), La Plata 1900, Argentina; 3 Consejo Nacional de Investigaciones Científicas y Tecnolíogicas (IFLP-CCT-CONICET)-C. C. 727, La Plata 1900, Argentina

**Keywords:** information geometry, disequilibrium, semi-classical descriptions

## Abstract

We associate here the relationship between de-coherence to the statistical notion of disequilibrium with regards to the dynamics of a system that reflects the interaction between matter and a given field. The process is described via information geometry. Some of its tools are shown here to appropriately explain the process’ mechanism. In particular we gain some insight into what is the role of the uncertainty principle (UP) in the pertinent proceedings.

## 1. Introduction

The quantum de-coherence concept arose in the early 1980s due to, among others, Zeh, Zurek, and Habib [1,2,3] A and has been exhaustively investigated in the literature. We re-examine it here from the viewpoint of information geometry and one of its central subjects, the disequilibrium *Q*, to show that *Q* grows as coherence augments.

Information geometry is a Riemannian geometric inquiry of statistical models represented by families of probability distributions. A statistical model portrays a manifold whose points are probability distributions from the model [4]. The well known Fisher information as a Riemannian metric on statistical manifolds [5]. We here compare the role played by Fisher’s information measure (FIM) with that of the much more recent consept of disequilibrium *Q*. Indeed, *Q* is the protagonist of the present study. We begin our proceedings with a brief *Q*-sketch.

### 1.1. Statistical Order and Disorder

Consider a continuous uniform probability. Each possible micro-state or phase-space cell is equally likely. We call this situation one of maximum disorder. The opposite scenario is that in which just ONE micro-state has probability unity, while the remaining ones have probability zero. We call this perfect order.

### 1.2. Statistical Quantifiers

Consider now the distribution function (PDF) f(x). Its associated Shannon information measure (Entropy) *S* is [6]

(1) S[f]=−∫fln(f)dx,

a global nature quantifier, not too sensitive to f—changes taking place on a small-sized region. The opposite instance is that of the above mentioned Fisher’s Information Measure (FIM) F [5], that measures the gradient content of *f* and is accordingly quite sensitive to tiny localized perturbations. It reads [5]

(2) $$F\left[f\right]\;=\;\int \;\left[\frac{|\vec{\nabla }{f|}^{2}}{f}\right]\;dx.$$

The FIM linked to translations of a one-dimensional observable *x* with corresponding probability density f(x) is [7]

(3) $$I_{x}=\int dx\;f\left(x\right)\;{\left(\frac{\partial \ln f(x)}{\partial x}\right)}^{2}\;dx,$$

ans obeys the so called Cramer Rao inequality (CRI)

(4) $${\left(\Delta x\right)}^{2}\geq I_{x}^{-1}$$

involving the variance of the stochastic variable *x* [7,8]

(5) $${\left(\Delta x\right)}^{2}=\langle x^{2}\rangle -{\langle x\rangle }^{2}=\int dx\;f\left(x\right)\;x^{2}-{\left(\int dx\;f(x)\;x\right)}^{2}.$$

Let $P=\{p_{i};\;i=1,\cdots ,N\}$ be a discrete probability distribution set for a system with *N* possible states [9,10,11] and references therein. Here we follow Ferri and coworkers [12] and cast FIM in the fashion

(6) $$F\left[P\right]\;=\frac{1}{4}\;\sum_{i=1}^{N-1}\;2\frac{{\left(p_{i+1}-p_{i}\right)}^{2}}{\left(p_{i+1}+p_{i}\right)}\;.$$

If our system lies in a rather ordered state, being represented by a narrow probability distribution function (PDF), we face a Shannon Entropy S∼0 and a FIM $F∼F_{max}$. On the other hand, in a very disordered state one looks at an almost flat PDF and F∼0 [13].

Related quantifiers are also the statistical complexity *C* and the disequilibrium *Q* [14,15,16,17,18]. *Q* is the (normalized) distance in probability space between the uniform distribution (maximum statistical disorder) and the extant one. The idea is to consider two opposite stages: (1) perfect order or (2) maximal randomness (no correlations at all) [14]. In between (1) and (2) multiple degrees of correlation might be present. In this instance, the notion of disequilibrium *Q* is, to repeat, a distance in probability space between the current probability distribution and the uniform one [15]. *Q* permits constructing a kind of hierarchy. If one encounters privileged states amongst the accessible ones. *Q* would then be maximal. Instead, in the case of the entropy things are exactly reversed. *S* is minimal for perfect order and maximal for total disorder. Thus, L. Ruiz, Mancini, and Calvet (LMC) [14] established the form for a statistical complexity *C* in the fashion

C=DS,

a functional of the probability distributions (PDs) that adequately grasp correlations in the way that entropy does so with randomness [14]. *Q* [14] adopts, for a system with *N* accessible states, the form

$$Q=\sum_{i=1}^{N}\;{\left(p_{i}-\frac{1}{N}\right)}^{2}.$$

Here, $p_{1},p_{2},\ldots ,p_{N}$ are the individual normalized probabilities ($\sum_{i=1}^{N}\;p_{i}=1$) [14]. *Q* attains the maximum value (unity) for a fully ordered state and vanishes in the case in which all $p_{i}$ are equal.

## 2. The Model to Be Scrutinized

One deals with a peculiar bipartite system that is concerned with the contribution of a strong external field’s zero-th mode to the emission of charged meson pairs [19,20,21]. The concomitant Hamiltonian is

(7) $$\hat{H}\;=\;\frac{1}{2}\left(\;\frac{{\hat{p}}^{2}}{m_{q}}\;+\;\frac{{P_{A}}^{2}}{m_{cl}}\;+\;m_{q}\omega^{2}{\hat{x}}^{2}\;\right).$$

In (7), (i) $\hat{x}$ and $\hat{p}$ are quantum operators, (ii) *A* and $P_{A}$ are classical canonical conjugate variables, and (iii) $\omega^{2}={\omega_{q}}^{2}+e^{2}A^{2}$ is an interaction term that introduces nonlinearity in the problem. $\omega_{q}$ is a frequency and *e* the electrical charge. Further, $m_{q}$ and $m_{cl}$ are masses corresponding to the quantum and classical systems. One sees in [20] that handling Equation (7) leads one to facing an autonomous system of nonlinear coupled equations. We write it below.

(8) $$\begin{array}{ccc} \frac{d\langle {\hat{x}}^{2}\rangle }{dt} & = & \frac{\langle \hat{L}\rangle }{m_{q}}\;,\\ \frac{d\langle {\hat{p}}^{2}\rangle }{dt} & = & -m_{q}\;\omega^{2}\langle \hat{L}\rangle \;,\\ \frac{d\langle \hat{L}\rangle }{dt} & = & 2\left(\frac{\langle {\hat{p}}^{2}\rangle }{m_{q}}-m_{q}\;\omega^{2}\langle {\hat{x}}^{2}\rangle \right)\;,\\ \frac{dA}{dt} & = & \frac{P_{A}}{m_{cl}}\;,\\ \frac{dP_{A}}{dt} & = & -e^{2}m_{q}\;A\langle {\hat{x}}^{2}\rangle \;. \end{array}$$

Above, $2\hat{L}=\hat{x}\hat{p}+\hat{p}\hat{x}$, involving the correlation operator $L=(\hat{x}\hat{p}+\hat{p}\hat{x})/2$. Equation (8) is obtained from Ehrenfest’s relations [20]. In order to treat the classical limit one must consider the classical counterpart of Equation (7)

(9) $$H\;=\;\frac{1}{2}\left(\frac{p^{2}}{m_{q}}\;+\;\frac{{P_{A}}^{2}}{m_{cl}}\;+\;m_{q}\omega^{2}x^{2}\right),$$

in which all variables are classical.

Of great importance for our present endeavor is the new quantity *I*, a motion-invariant [20,21] that emerges from the system of Equation (8). *I* is closely related to the Uncertainty Principle, as one realizes from its definition

(10) $$I\;=\;\langle {\hat{x}}^{2}\rangle \langle {\hat{p}}^{2}\rangle -\frac{{\langle \hat{L}\rangle }^{2}}{4}\geq \frac{\hbar^{2}}{4}.$$

A classical evaluation of *I* leads to

$$I=x^{2}p^{2}-L^{2}/4\equiv 0.$$

Using Hamilton’s classical equations one sees that the classical counterpart of (8) is a set of equations that look identical to (8) if one replaces quantum mean values with classical variables. For example, $\langle {\hat{x}}^{2}\rangle \Rightarrow x^{2}$, $\langle {\hat{p}}^{2}\rangle \Rightarrow p^{2}$ and $\langle \hat{L}\rangle \Rightarrow L=2xp$.

One moves towards the classical limit by making I→0 o, also, via a quantity called the “relative energy” *E*

(11) $$E_{r}\;=\;\frac{E}{I^{1/2}\omega_{q}}\to \infty ,$$

($E_{r}\geq 1$), with *E* the total energy of the system. Here we employ suitable arbitrary units and set

$$\omega_{q}=1,\;\mathrm{in}\;\;\;\mathrm{arbitrary}\;\;\;\mathrm{frequency}\;\;\;\mathrm{units}.$$

Also (in pertinent accompanying units) we need

$$E=0.6,\;m_{cl}=m_{q}=1,\;A=1.$$

The charge is e=1, also in suitable units. We let *I* vary in the range 0<I<∞. The degree of convergence between classical and quantum results from the limit $E_{r}\to \infty$ of Equation (11) can be determined by the norm N of the vector $\Delta u=u-u_{cl}$ [20],

(12) $$N_{\Delta u}=\left|u-u_{cl}\right|.$$

We have a three components vector $u=(\langle {\hat{x}}^{2}\rangle ,\langle {\hat{p}}^{2}\rangle ,\langle \hat{L}\rangle )$ that constitutes the “quantum” part of the solution of the system (8) and also a vector $u_{cl}=\left(x^{2},p^{2},L\right)$, its classical counterpart. We stress that the classical counterpart of Equation (8) entails I=0.

One faces here a system interesting for both Quantum Optics and Condensed Matter, particularly because one deals with a bosonic system that possesses quasi-periodic and unbounded regimes, separated by an unstable region. This fact makes the interaction with a classical mode a quite interesting phenomenon. The model displays a specially complex sub-regime, with superposition of both chaos and complexity. One finds strong correlation between classical and quantum degrees of freedom. The present model has been detailedly investigated in [20]. A few words are in order here in connection with the meaning of our all important quantifier *I*, an invariant of the motion closely related to the uncertainty principle. It measures uncertainty. The smaller *I*, the more classical the composite system becomes. This is our view of classic structure here. It intensifies whenever *I* diminishes. Paradoxically, if it diminishes to a sufficient degree, chaos ensues. Contrarily, whenever *I* sufficiently grows, chaos dies.

One sees there plotted diverse dynamical quantities as a function of $1<E_{r}<\infty$, which display a typical decoherence process.

### Three Zones for Our Process

Three $E_{r}$-regions are to be distinguished

1. quantum,
2. transitional (semi-classical), and
3. classical,

that characterize the de-coherence process (DC). Quite importantly, this DC can be described by *I*. As *I* grows, de-coherence diminishes. This *I*-described de-coherence picture shows that, in some special *I*-sub-zone, chaos is always found. The relative number of chaotic orbits (with respect to thetotal number of orbits) grows as de-coherence intensifies itself. The associated orbits display features that can not be described using the global measure Equation (12). One needs for the purpose a local measure like FIM. In this work we investigate the performance of *Q* as local measure.

We will focus on the coherence notion. In de-cohering proceedings, one can describe the process in terms of disequilibrium *Q* versus *I* plots and compare such description with one in terms of FIM versus *I*. As important features we notice that [21]

- At a low *I* value, $I=I_{P}=0.0325$, chaos ensues.
- At a much lower value $I=I_{class}=7.75\exp\left(-4\right)\approx 0.001$, the classical zone begins to emerge and continues till we reach I=0. Accordingly,
- For $I\leq I_{class}$ we are entirely within the classical zone (maximum decoherence).
- The transition zone corresponds to the range $I_{class}\leq I\leq I_{P}$.
- For $I\geq I_{P}$ one reaches the quantum kingdom (minimum de-coherence).

## 3. An Essential Task: Determining the Underlying Probability Distribution

Our proceedings are statistical in nature. One necessitates then an adequate probability distribution. To such an end we appeal to a standard approach to determine the proper probability distribution function *P* associated to our above described dynamical system (DS). The DS is first of all translated into a time series (in the present instance, an *I*-series). The concomitant process is described in [22,23,24,25,26,27]. We specialize here in the ordinal-patterns methodology of Bandt and Pompe (BP) [28]). The BP technique yields a probability distribution out of our i—series, derived as the solutions of Equation (8). From them we extract the values of $<x^{2}>$, one of them for each *I*-value. Let is insist on the fact that these values of $<x^{2}>$ constitute a time-series.

We can get the looked for probability distribution *P* once we fix the so-called BP embedding dimension *D* and the so called BP time delay τ [28]. Such methodology has been detailedly explained and used in [21,29,30]).

## 4. Our Present Results

The present data, that will enter the Bandt-Pompe procedure, emerge as the solutions of (8). From them we obtain the values of $\langle x^{2}\rangle$. So as to assess our results remember that the disequilibrium *Q* varies in the range

0≤Q≤1,

so that unity signals maximum statistical order.

We describe now our results so as to build up an scenario based on displays as a function of *Q*. As for the initial conditions that the task requires we varied *I* so as to obtain our different *Q* values.

### 4.1. Quantum Uncertainty *I* Versus Statistical Order *Q*

Let us begin by differentiating the meanings of the quantifiers *Q* and *I*. For both, coherence augments as the quantifiers grow.

- Remember that *Q* measures statistical order. Its value depends only on the extant probability distribution (PD), extracted here from a time series via the Bandt Pompe technique.
- In turn, the quantum uncertainty (QU) we deal with here derives directly from our non lineal system of motion-equations. The motion equations involve mean values that use amplitudes (wave functions). Classically, QU vanishes, of course. QU is measured by our variable *I*.

### 4.2. Our Plots

**Figure 1:**
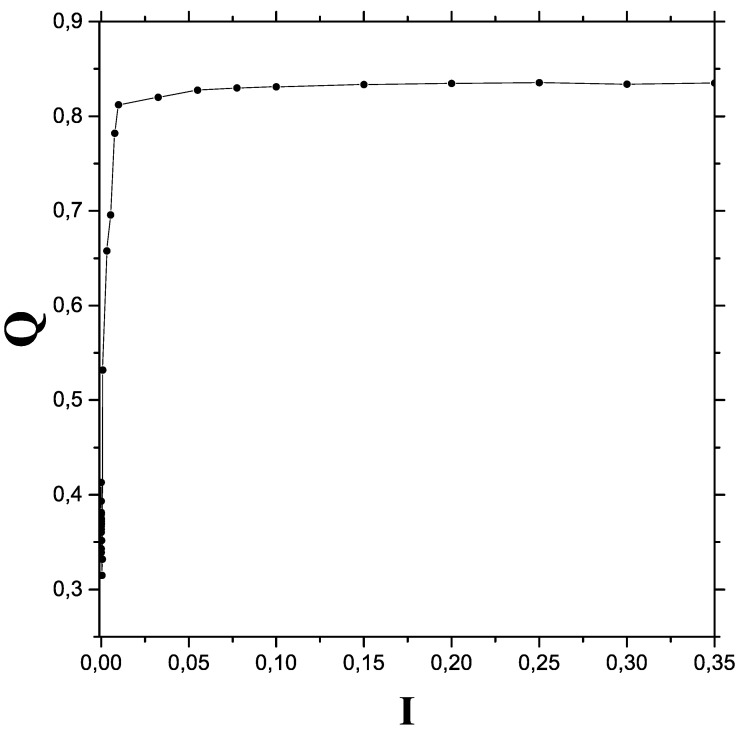
The quantum-classical transition process as viewed from a *Q* versus *I* viewpoint. Our rater ample I—range encompasses classical, semi-classical, and quantum regions.

Figure 1 depicts the disequilibrium *Q* versus *I* in a suitable I—range, according to the I—itemizing displayed above (0<I<0.3). We consider 5000 data-points per initial condition and take 41 different values of *I*. Pure classic structure is seen ar the extreme left with a very low *Q* value. A process of disequilibrium quasi-linear growth characterizes the transition zone, that leads the quantum realm with constant *Q*.

**Figure 2:**
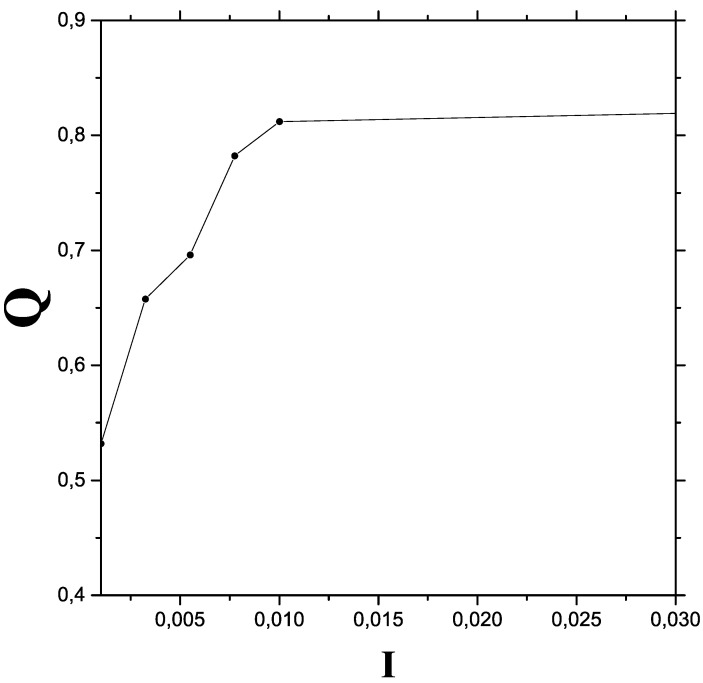
The quantum-classical process (transition region that encompasses chaos): *Q* vs. *I*.

In Figure 2 we consider a more limited I—range in order to appreciate more details of the transition region.

**Figure 3:**
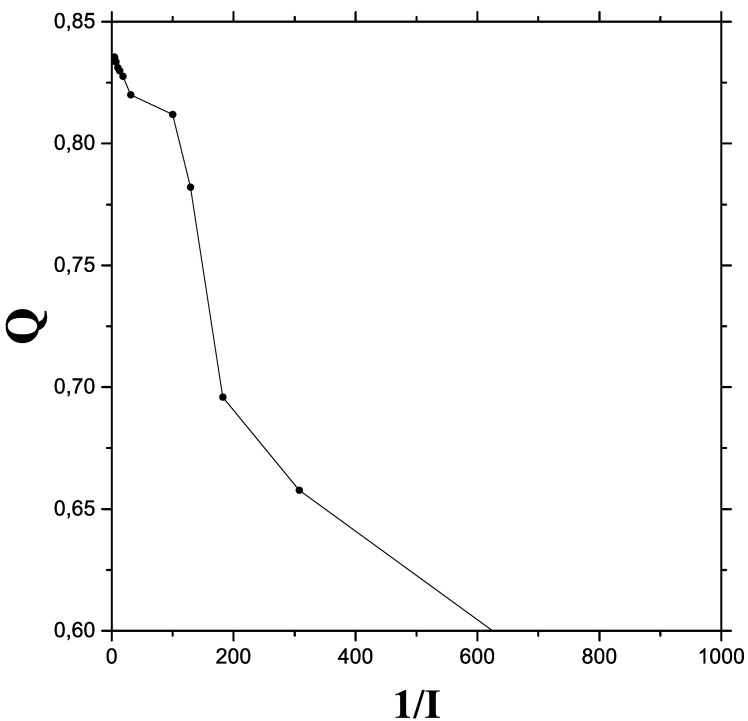
The quantum-classical process viewed from plotting *Q* vs. 1/I.

Figure 3 reiterates the message of the previous two graphs by plotting *Q* versus 1/I.

**Figure 4:**
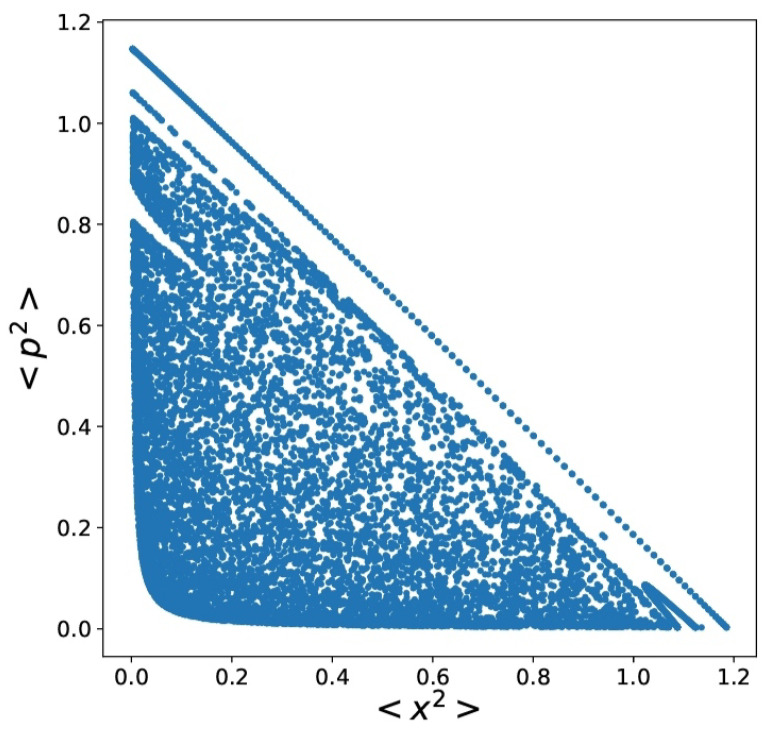

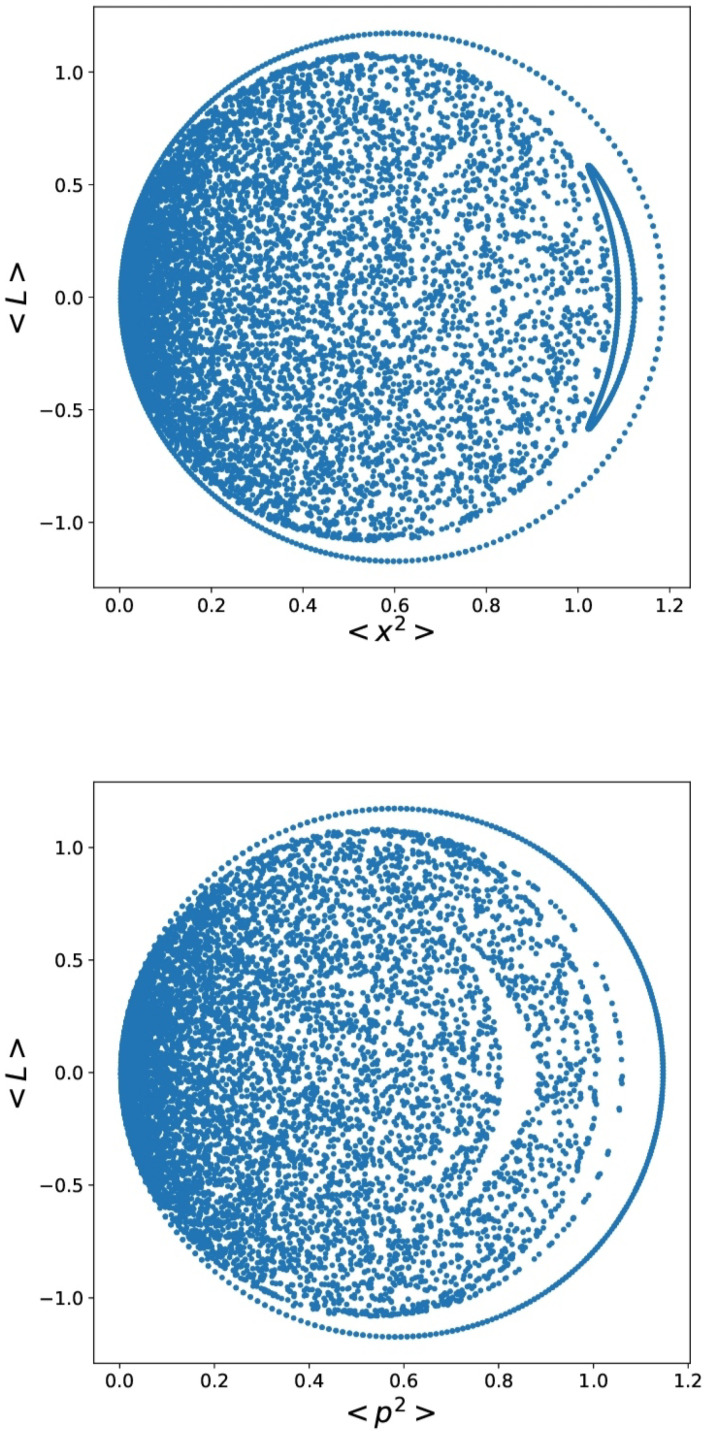
A=0 Poincaré surfaces for E=0.6, A(t=0)=0, $m_{q}=m_{cl}=\omega_{q}=e=1$. $I=0.003>I_{class}$.

The next graph (Figure 4) illustrates how chaos ensues by plotting Poincaré surfaces.

**Figure 5:**
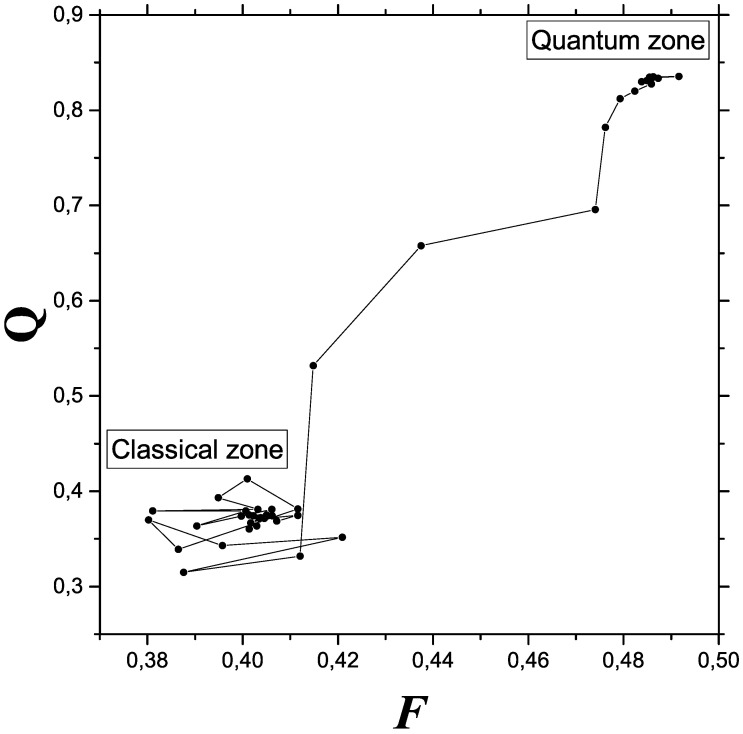
Details of the classical-quantum transitional are best appreciated in this plot FIM versus *Q*. In both Fisher terms and disequilibrium ones, the quantum-classical changeover can be seen as an order (quantum)—disorder (classical, including chaos) transition.

Plotting Fisher’s information measure (FIM) *F* versus *Q* in an appropriate range allow one to envisage, in Figure 5, details of the classical-quantum transition (CQ) not easily available without Fisher information’s help. The transition process consist in gaining order (represented by both FIM and *Q*) in a peculiar fashion. The CQ changeover is seen as a disorder-order process.

## 5. Conclusions

We have here visualized the classical-quantum (CQ) transition as a process of disorder-order change. Both Fisher’s information (FIM) and disequilibrium *Q* grow in such process from the classical kingdom to the quantum one.

Our results refer to to two kinds of probabilities that describe our model. One is that distribution extracted from a time series (indeed, an *I* series) by the Bandt Pompe statistical procedure. The other probability-elemnts derive from the non linear set of equaions of motions, that provide us with expectation values (e.g, $<x^{2}>$).

The features of the CQ route from, let us repeat, are depicted via (1) the motion invariant *I*, and upper bound to the quantum uncertainty, (2) the Fisher information measure (FIM), and (3) the disequilibrium *Q*. Our present results are in entire accordance with those of [31,32,33].

As far as we know, the present link between order.disorder and entropy has been investigated only here. We are now extending the treatment to Tsallis’ and Renyi entropies.

## Data Availability

Not applicable.
